# Therapeutic targeting of polo-like kinase 1 using RNA-interfering nanoparticles (iNOPs) for the treatment of non-small cell lung cancer

**DOI:** 10.18632/oncotarget.2664

**Published:** 2015-01-06

**Authors:** Joshua A. McCarroll, Tanya Dwarte, Huricha Baigude, Jason Dang, Lu Yang, Rafael B. Erlich, Kathleen Kimpton, Joann Teo, Sharon M. Sagnella, Mia C. Akerfeldt, Jie Liu, Phoebe A. Phillips, Tariq M. Rana, Maria Kavallaris

**Affiliations:** ^1^ Children's Cancer Institute, Lowy Cancer Research Centre, Randwick, UNSW Australia (UNSW), NSW, Australia; ^2^ ARC Centre of Excellence in Convergent Bio-Nano Science and Technology, Australian Centre for NanoMedicine, UNSW, NSW, Australia; ^3^ Pancreatic Cancer Translational Research Group, Lowy Cancer Research Centre, Prince of Wales Clinical School, UNSW, NSW, Australia; ^4^ Program for RNA Biology, Sanford-Burnham Medical Research Institute, La Jolla, CA, USA; ^5^ Department of Pediatrics, University of California, San Diego, School of Medicine, La Jolla, CA, USA

**Keywords:** Non-small cell lung cancer, Interfering nanoparticles, Polo-like kinase 1, siRNA, Orthotopic non-small cell lung cancer mouse model

## Abstract

Non-small cell lung cancer (NSCLC) remains the most common cause of cancer death worldwide due its resistance to chemotherapy and aggressive tumor growth. Polo-like kinase 1 (PLK1) is a serine-threonine protein kinase which is overexpressed in cancer cells, and plays a major role in regulating tumor growth. A number of PLK1 inhibitors are in clinical trial; however, poor tumor bioavailability and off-target effects limit their efficacy. Short-interfering-RNA (siRNA) holds promise as a class of therapeutics, which can selectively silence disease-causing genes. However, siRNA cannot enter cells without a delivery vehicle. Herein, we investigated whether RNAi-interfering nanoparticles could deliver siRNA to NSCLC cells and silence PLK1 expression *in vitro* and *in vivo*. iNOP-7 was non-toxic, and delivered siRNA with high efficiency to NSCLC cells. iNOP-7-PLK1 siRNA silenced PLK1 expression and reduced NSCLC growth *in vitro*. Notably, iNOP-7 delivered siRNA to orthotopic lung tumors in mice, and administration of iNOP-7-PLK1 siRNA reduced lung tumor burden. These novel data show that iNOP-7 can deliver siRNA against PLK1 to NSCLC cells, and decrease cell proliferation both *in vitro* and *in vivo*. iNOP-7-PLK1 siRNA may provide a novel therapeutic strategy for the treatment of NSCLC as well as other cancers which aberrantly express this gene.

## INTRODUCTION

Lung cancer is a lethal adult cancer accounting for the most cancer deaths worldwide [1, 2]. In 2010, lung cancer was the leading cause of death in men, and the second leading cause of death in women [1, 2]. The most common form, Non-Small Cell Lung Carcinoma (NSCLC) accounts for greater than 80% of all cases [1]. Over half of NSCLC patients have developed metastases at the time of diagnosis and 5-year survival is only ~14% [1]. Hence, there is an urgent need to develop novel treatment strategies to combat this disease.

Anti-mitotic drugs (taxanes, *vinca alkaloids*, epothilones) which target microtubules (cytoskeletal proteins that comprise α- and β-tubulin heterodimers) are an important component in the treatment of many cancers but, are associated with resistance and toxic side effects [3]. Recently, new-generation anti-mitotic agents which target mitotic kinases have received attention [4]. For instance, polo-like protein kinase 1 (PLK1) is a serine/threonine kinase which belongs to the polo-like kinase protein family (contains 5 members, PLK1-5). PLK1 is the most studied PLK protein, and is essential in regulating different stages of the cell cycle including mitotic entry via phosphorylation of cyclin B1 and Cdc25c, spindle formation, chromosome segregation and cytokinesis [5]. Importantly, PLK1 is highly expressed in different tumor types including NSCLC, and is often correlated with aggressive disease and poor survival [5, 6]. More recently, PLK1 has also been shown to contribute to tumor growth via mechanisms that are independent of the cell cycle [7, 8]. Several studies have also demonstrated a role for PLK1 in promoting the growth and survival of cancer stem cells [9, 10]. Based on strong preclinical data for PLK1 inhibition and decreased cancer cell growth, several PLK1 chemical inhibitors are in clinical trial [4]. However, several limitations have emerged such as, lack of target specificity, off-target side effects (inhibition of other PLK proteins) and poor tumor bioavailability [11, 12]. For instance, the PLK1 inhibitor BI2536 was shown to arrest the growth of primary cardiac fibroblasts isolated from rats, suggesting potential cardiac off-target toxicity [13]. Another study showed that BI2536 had limited efficacy on tumor growth in a hepatocellular carcinoma mouse model, which had a tumor microenvironment which closely mimics the human setting [11]. This was attributed to low intratumoral levels of BI2536 which led to resistance [11].

RNA interference (RNAi) is a gene-silencing mechanism which occurs in mammals and can be activated in cells by introducing chemically synthesized short-interfering RNA (siRNA) [reviewed in [14]]. An advantage of this mechanism is its potency, and potential to silence genes that are considered to be ‘non-druggable’. However, before siRNA can be used clinically, a delivery vehicle is required due to the net negative charge of siRNA preventing its entry into cells as well as potential for rapid degradation by RNAases present in serum and elimination by the reticulo-endothelial system [14]. To overcome these obstacles, non-viral nanoparticles are often used to encapsulate siRNA to protect it from degradation and facilitate delivery to the cell cytoplasm via passive or active targeting mechanisms [15]. Importantly, nanoparticles have already been used to deliver siRNA to silence key genes that promote human disease in preclinical animal models, and are in human clinical trial [16–18]. However, despite their promise, there is still a need to develop nanoparticle systems which can be readily synthesized in large-scale amounts for clinical translation, and possess physicochemical properties that allow for easy self-assembly with siRNA and the conjugation of cancer-cell targeting moieties to their surface.

We previously reported on the design and synthesis of a novel non-viral nanoparticle as a delivery vehicle for RNAi molecules [19–22]. Interfering Nanoparticle-7 (iNOP-7) is a highly branched generation four poly-L-lysine dendrimer with 32 amino groups on its surface [19, 20]. The amino acid groups are positively charged under physiological conditions thus allowing it to rapidly self-assemble with siRNA or other negatively charged molecules. In addition, the amino groups on the surface of iNOP allow for chemical conjugation of various moieties. For instance, iNOP-7 was conjugated with 7 oleic acid lipid chains to increase hydrophobicity, reduce toxicity and increase cell uptake [21]. iNOP-7 is 14nm in size, and when complexed to siRNA increases to approximately 180nm [20]. In mice, systemic administration of iNOP-7 complexed to siRNA silenced the expression of a target gene in the liver which is involved in regulating cholesterol metabolism [19]. The versatility of iNOP-7 as a delivery vehicle for RNAi molecules was illustrated when it also delivered an anti-miRNA single-stranded oligonucleotide inhibitor targeted to miR-122 [22]. This miRNA is expressed at high levels in the liver, and plays a critical role in regulating the expression of a collection of genes which modulate cholesterol metabolism. Delivery of iNOP-7-anti-miR-122 complexes to mice silenced the expression of miR-122 levels in the liver by >80% and led to the alteration in expression of genes involved in cholesterol metabolism, resulting in a 30% reduction of total cholesterol [22]. Notably, iNOP-7 was non-toxic and non-immunostimulatory both *in vitro* and *in vivo* [19, 22]. However, whether iNOP-7 can be used to deliver siRNA to tumor cells is unknown.

In this study, we used iNOP-7 to complex and deliver siRNA targeted against PLK1 to silence its expression in multiple NSCLC cell lines. Silencing PLK1 expression using iNOP-7-PLK1 siRNA led to a marked decrease in NSCLC cell proliferation. This correlated with a strong induction of apoptosis. Moreover, we demonstrated for the first time that iNOP-7 could deliver clinically-relevant amounts of PLK1 siRNA to lung tumors and reduce their proliferation in an orthotopic NSCLC mouse model which closely mimics the tumor microenvironment observed in the clinical setting.

## RESULTS

### Polo-like kinase 1 (PLK1) is highly expressed in NSCLC cells

**Figure 1:**
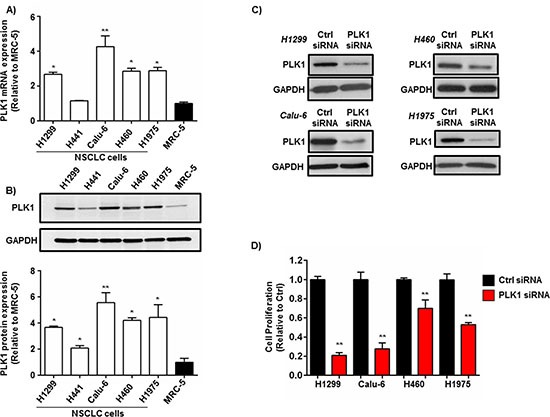
PLK1 expression in NSCLC cells and the effect of PLK1 knockdown on NSCLC cell proliferation **(A)** qPCR analysis showing a increase in PLK1 mRNA expression in NSCLC cells (H1299, H441, Calu-6, H460, H1975) vs. normal human lung fibroblasts (MRC-5), n = 3 independent experiments; bars, mean ± SE. **p < 0.001, *p < 0.01. (**B)** Representative western blot and densitometry graph demonstrating a significant increase in PLK1 protein levels in NSCLC cells (H1299, H441, Calu-6, H460, H1975) vs. normal human lung fibroblasts (MRC-5), n = 3 independent experiments; bars, mean ± SE. **p < 0.001, *p < 0.01. GAPDH was used a protein loading control. **(C)** Representative western blots showing a reduction of PLK1 protein expression in NSCLC cells (H1299, H460, Calu-6, H1975) 72h post-transfection with PLK1 siRNA (100 nM) complexed to lipofectamine 2000 (L2K). Cells treated with non-functional (Ctrl) siRNA served as controls. GAPDH was used a protein loading control. **(D)** Cell proliferation assay showing a significant reduction in NSCLC (H1299, H460, Calu-6, H1975) cell proliferation 72h post-transfection with PLK1 siRNA (100 nM) complexed to L2K. Cells treated with non-functional (Ctrl) siRNA served as controls, n = 3; bars, mean ± SE. **p < 0.01.

To assess PLK1 levels in NSCLC cells, the gene and protein expression of PLK1 was measured by qPCR and western blotting in 5 different NSCLC cell lines derived from primary and metastatic sites. Moreover, these cell lines were chosen based on their expression of genetic alterations (KRAS, p53 and EGFR mutations) which are clinically relevant and represent the heterogeneity of the disease [23]. PLK1 mRNA expression was significantly increased [2-4 fold increase (*p* < 0.01)] at the gene level in 4 out of 5 NSCLC cell lines when compared to normal human (non-tumorigenic) lung fibroblasts (MRC-5) (Figure 1A). PLK1 protein expression was also significantly increased [2-6 fold increase (*p* < 0.01)] in NSCLC cells when compared to normal lung fibroblasts (Figure 1B).

### Silencing PLK1 expression using siRNA reduces NSCLC cell proliferation and viability *in vitro*

To examine whether PLK1 is a strong candidate for RNAi in NSCLC cells, we tested the gene silencing efficacy of 2 individual PLK1 siRNA sequences which target different regions of the PLK1 gene in H1299 cells. Both siRNAs (PLK1-siRNA #1 and PLK1-siRNA #2) potently silenced PLK1 mRNA expression in H1299 cells by >80% (*p* < 0.001), 48h post-transfection when complexed to the commercial transfection agent Lipofectamine 2000 (L2K) (Supplementary Figure 1). Next, we assessed the effect of silencing PLK1 expression on NSCLC cell proliferation. Four different NSCLC cell lines (H1299, H460, Calu-6 and H1975) were transfected with PLK1 siRNA (100 nM) complexed to L2K. Seventy-two hours post-transfection cell lysates were collected and PLK1 expression measured by western blotting. PLK1 protein expression was reduced in all 4 NSCLC cell lines compared to controls (Figure 1C). Furthermore, knockdown of PLK1 significantly inhibited cell proliferation in all 4 NSCLC cell lines (Figure 1D). Notably, cell growth was reduced by >70% (*p* < 0.001) in both H1299 and Calu-6 NSCLC cells when compared to controls (Figure 1D). The potent reduction in cell proliferation following PLK1 gene silencing (100 nM siRNA) was further validated in both the H1299 and Calu-6 cell lines using 2 individual PLK1 siRNAs at different low concentrations (1-25 nM) (Supplementary Figure 2). Indeed, treatment with as little as 1 nM of PLK1 siRNA was able to reduce PLK1 protein expression and cell proliferation in both H1299 and Calu-6 NSCLC cell lines when compared to controls (Supplementary Figure 2A-D).

**Figure 2:**
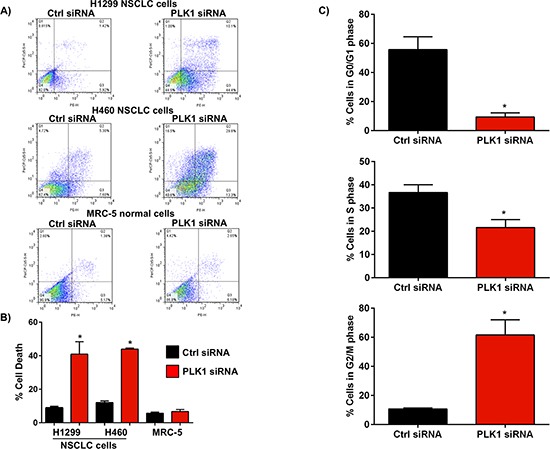
Effect of PLK1 knockdown using siRNA on NSCLC cell death and cell cycle progression **(A & B)** Representative flow cytometry plots of annexin V staining in NSCLC cells (H1299, H460) and a graph showing an increase in the percentage of cells which stained positive with annexin V, 72h post-transfection with PLK1 siRNA (100 nM) complexed to lipofectamine 2000 (L2K). Cells treated with non-functional (Ctrl) siRNA served as controls. No difference in annexin V staining was observed in normal human lung fibroblasts (MRC-5), 72h post-transfection with PLK1 siRNA complexed to L2K when compared to controls (Ctrl siRNA), n = 3; bars, mean ± SE. **p < 0.01. **(C)** Cell cycle analysis shows a significant increase in G2/M cell cycle arrest in NSCLC cells, 48h post-treatment with PLK1 siRNA complexed to L2K. Cells treated with non-functional (Ctrl) siRNA served as controls, n = 3; bars, mean ± SE. **p < 0.01.

Inhibition of PLK1 has been reported to induce apoptosis in a number of different types of cancer cells via a G2/M cell cycle arrest [5]. To confirm whether the observed decrease in cell proliferation in NSCLC cells following treatment with PLK1 siRNA was associated with increased cell death and/or cell cycle arrest, we treated 2 different NSCLC cell lines (H1299 and H460) with PLK1 siRNA complexed to Lipofectamine 2000 (L2K), and measured apoptosis by annexin V staining and flow cytometry. Cell cycle distribution was also measured by propidium iodide staining and flow cytometry 48h post-PLK1 siRNA transfection. Silencing PLK1 expression using siRNA markedly increased cell death in both H1299 and H460 NSCLC cells, 72h post-transfection when compared to cells treated with control siRNA (Figure 2A and 2B). The increase in cell death correlated to a strong induction in G2/M cell cycle arrest 48h post-treatment (Figure 2C). Interestingly, silencing PLK1 expression using siRNA in normal human lung fibroblasts (MRC-5) did not induce cell death (Figure 2A and 2B and Supplementary Figure 3). This suggests that PLK1 may be playing an important role in regulating NSCLC cell survival. Collectively, these results provide strong evidence that PLK1 is highly expressed in NSCLC cells, and that silencing its expression using siRNA strongly inhibits cell proliferation via an induction of mitotic arrest and cell death.

### iNOP-7 delivers siRNA to NSCLC cells *in vitro*

**Figure 3:**
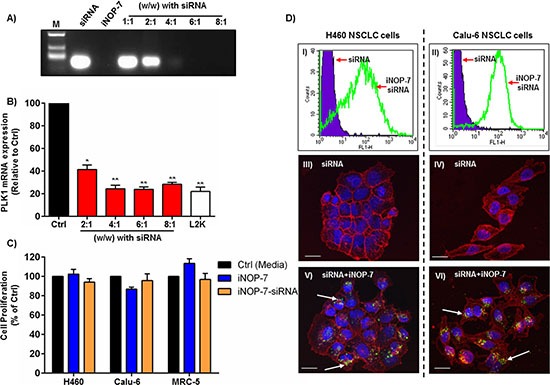
iNOP-7 siRNA delivery to NSCLC cells *in vitro* **(A)** Representative agarose gel showing free unmodified siRNA (siRNA, 60 ng) alone (lane 2), iNOP-7 alone (lane 3), or increasing amounts of iNOP-7 complexed to siRNA (1:1-8:1 w/w ratio with siRNA) (lanes 5-9). M = molecular weight marker. **(B)** A graph showing PLK1 knockdown in H1299 cells, 24h post-transfection with increasing amounts of iNOP-7 complexed to PLK1 siRNA (100 nM). Cells incubated in culture media or transfected with PLK1 siRNA complexed to lipofectamine 2000 (L2K) served as controls, n = 3; bars, mean ± SE. *p < 0.01, **p < 0.001. **(C)** A graph showing no significant toxicity in NSCLC cells (H460, Calu-6) or normal human fibroblasts (MRC-5) when treated with iNOP-7 alone or complexed to non-silencing siRNA (8:1 w/w) 24h post-transfection. Cells incubated in culture medium served as control, n = 3; bars, mean ± SE. **(D)** Flow cytometry histograms (I and II) and confocal images (III-VI) demonstrating cell uptake of fluorescently labeled-siRNA (green) in NSCLC (H460 and Calu-6) cells complexed to iNOP-7 (8:1 w/w) 24h post-transfection. Fluorescent siRNA (Green), cell membrane (Red), and nuclear DNA (Blue), n = 3 (white arrows show the location of siRNA).

In the preceding section our data strongly supports PLK1 siRNA inhibition as a potential therapeutic strategy against NSCLC cells. However, an effective siRNA delivery vehicle is required. We previously reported on the synthesis of a novel modified dendrimer nanoparticle (iNOP-7) which could deliver RNAi molecules to the liver of mice and silence the expression of key genes or miRNAs involved in regulating cholesterol metabolism [19–22]. However, whether iNOP-7 could deliver siRNA to tumor cells *in vitro* and *in vivo* was unknown. First, we examined how much iNOP-7 was required to fully self-assemble with siRNA via an electrostatic interaction using agarose gel electrophoresis. Non-complexed siRNA migrated to the bottom of the gel (Figure 3A). In contrast, increasing amounts of iNOP-7 (2:1-8:1 w/w) complexed to siRNA prevented its migration, confirming our previous studies showing that upon simple mixing iNOP-7 was able to rapidly complex with siRNA [19–21] (Figure 3A). It was evident from these results that at 2:1 (w/w) iNOP-7 could complex approximately 50% siRNA, while at the higher amounts of 4:1-8:1 (w/w) iNOP-7 fully complexed siRNA (Figure 3A). To examine whether the differing iNOP-7-siRNA ratios possessed gene silencing activity, NSCLC cells were transfected with increasing amounts of iNOP-7 [2:1-8:1 (w/w)] complexed to a fixed amount of PLK1 siRNA (100 nM). Cells treated with PLK1 siRNA complexed to lipofectamine 2000 (L2K) or culture medium alone served as controls. iNOP-7 complexed to PLK1 siRNA at all of the chosen ratios [2:1-8:1 (w/w)] displayed gene silencing activity in NSCLC cells (Figure 3B). Notably, iNOP-7-PLK1 siRNA at the ratios of 4:1-8:1 (w/w) silenced PLK1 gene expression by >70% (*p* < 0.001), which was comparable to L2K (Figure 3B). Based on these results iNOP-7 at an 8:1 (w/w ratio with siRNA) was further characterized as a siRNA delivery vehicle for NSCLC cells.

In order to assess whether iNOP-7 at a 8:1 (w/w) ratio was non-toxic to cells, 2 different NSCLC cell lines (H460 and Calu-6) and normal human lung fibroblasts (MRC-5) were treated with iNOP-7 alone or complexed to non-functional siRNA. Seventy-two hours post-transfection cell viability was measured using colorimetric cell viability assays. Both iNOP-7 alone and iNOP-7-siRNA [8:1 (w/w)] were non-toxic in the 2 NSCLC cell lines and normal human lung fibroblasts (Figure 3C). To determine the cellular uptake and pattern of distribution of siRNA when complexed to iNOP-7, we treated 2 different NSCLC cell lines (H460 and Calu-6) with fluorescently labeled siRNA complexed to iNOP-7 [8:1 (w/w)]. Cells transfected with fluorescently labeled siRNA alone served as controls. Twenty-four hours post-transfection cell uptake was examined by flow cytometry and confocal microscopy. As expected siRNA alone was unable to enter the cells (Figure 3D, panels I-IV). In contrast, fluorescent siRNA complexed to iNOP-7 was able to efficiently enter both NSCLC cell lines as evidenced by a significant shift in green fluorescence (Figure 3D, panels I-II). Confocal microscopy demonstrated that fluorescent siRNA delivered by iNOP-7 was localized to the perinuclear regions of both cell lines (Figure 3D, panels V and VI). Finally, to track the internalization and release of siRNA from lysosomes and endosomes within the cell cytoplasm, iNOP-7-fluorescent siRNA (green) was transfected into NSCLC cells using iNOP-7 [8:1 (w/w)], and 1h, 4h and 18h post-transfection cells were stained with LysoTracker (red) to stain endosomes/lysosomes. One hour post-transfection some of the siRNA could be seen to co-localize with lysosomes/endosomes as indicated by the presence of yellow dots within the cell (Supplementary Figure 4). However, at 4h and 18h post-transfection, no co-localization of the siRNA with lysosomes/endosomes was observed (Supplementary Figure 4). This indicates that the siRNA was efficiently released into the cell cytoplasm following delivery by iNOP-7. Taken together, iNOP-7 was able to easily and rapidly self-assemble with siRNA, was non-toxic to cells, and could effectively deliver siRNA to NSCLC cells.

### iNOP-7-PLK1 siRNA silences PLK1 expression and induces apoptosis in NSCLC cells

**Figure 4:**
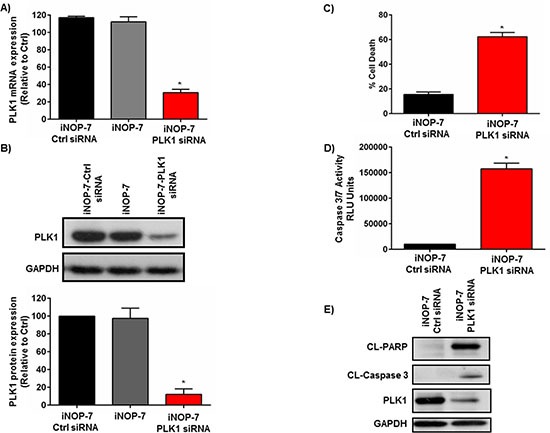
Effect of iNOP-7-PLK1 siRNA on NSCLC cell viability *in vitro* **(A)** A graph showing potent knockdown of PLK1 gene expression in H1299 cells transfected with iNOP-7-PLK1 siRNA (8:1 w/w) 48h post-transfection. Cells treated with iNOP-7 complexed to non-functional (Ctrl) siRNA or iNOP-7 alone served as controls, n = 3; bars, mean ± SE. *p < 0.001. **(B)** Representative western blot and densitometry graph demonstrating a significant decrease in PLK1 protein expression in H1299 cells, 72h post-transfection with iNOP-7-PLK1 siRNA (8:1 w/w). Cells treated with iNOP-7-non-functional (Ctrl) siRNA or iNOP-7 alone served as controls, n = 3; bars, mean ± SE. *p < 0.001. **(C)** Graph showing a significant increase in cell death in NSCLC cells 72h post-transfection with iNOP-7-PLK1 siRNA (8:1 w/w). Cells treated with iNOP-7-non-functional siRNA (Ctrl) siRNA served as controls, n = 3; bars, mean ± SE. *p < 0.001. **(D and E)** Representative graph and western blots demonstrating a significant increase in cleaved caspase 3/7 activity as well as cleaved PARP expression in NSCLC cells, 48h post-treatment with iNOP-7-PLK1 siRNA (8:1 w/w). Cells treated with iNOP-7-non-functional (Ctrl) siRNA served as controls. GAPDH was used as a protein loading control, n = 3; bars, mean ± SE. *p < 0.001.

Given that iNOP-7-siRNA could enter NSCLC cells with a high degree of efficiency, we then determined whether iNOP-7-PLK1 siRNA could potently silence PLK1 expression and induce apoptosis in NSCLC cells. H1299 NSCLC cells were transfected with iNOP-7-PLK1 siRNA (8:1 w/w). Cells treated with iNOP-7 (8:1 w/w) alone or iNOP-7-non-functional siRNA (8:1 w/w) served as controls. iNOP-7-PLK1 siRNA potently silenced PLK1 gene expression by >70% (*p* < 0.001), 48h post-transfection when compared to controls (iNOP-7 alone and iNOP-7-Ctrl siRNA) (Figure 4A). This correlated with a 90% (*p* < 0.001) decrease in PLK1 protein expression, 72h post-transfection (Figure 4B). Notably, treatment of NSCLC cells with iNOP-7-PLK1 siRNA induced a marked increase in cell death, 72h post-treatment as assessed by Annexin V staining and flow cytometry (Figure 4C). Moreover, the increase in cell death was associated with caspase 3/7-induced apoptosis as evidenced by a significant increase in caspase 3/7 activity and cleaved PARP expression 48h post-transfection (Figures 4D and 4E).

### iNOP-7 delivers siRNA to orthotopic NSCLC tumors *in vivo*

**Figure 5:**
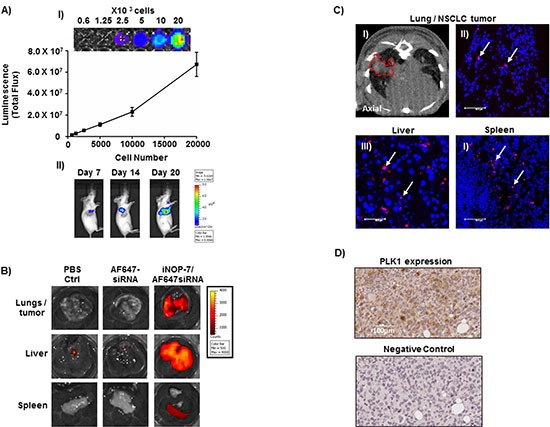
Establishment of a NSCLC bioluminescent mouse model and iNOP-7 siRNA delivery *in vivo* **(A)** Panel I, a representative image and graph showing increased luciferase activity with increasing H1299-Luc NSCLC cell numbers, n = 3 experiments. Panel II, pseudocolor images of mice injected orthotopically with H1299-Luc cells showing an increase in lung bioluminescence at days 7-20 post-tumor cell injection. **(B)** Representative *ex-vivo* fluorescent images of lungs and lung tumors, liver and spleen collected from mice with H1299-Luc orthotopic lung tumors, 4h post-injection (intravenous) with fluorescent siRNA (Red) complexed to iNOP-7. Mice injected with iNOP-7-fluorescent siRNA showed high fluorescence in the lung and lung tumors, liver and spleen. Mice injected with fluorescent siRNA alone or PBS served as controls. **(C)** Panel I, micro-CT image (axial) showing the presence of a growing tumor within the lungs of a mouse prior to injection with iNOP-7-fluorescent siRNA (red-dotted line marks the area of the tumor within the lung). Panels II-IV, are representative confocal microscopy images of frozen sections of lung tumor, liver and spleen showing the presence of fluorescent siRNA when delivered by iNOP-7, 4h post-injection (white arrows mark the location of fluorescent siRNA). **(D)** Panel I, Immunohistochemical image demonstrating PLK1 expression in orthotopic H1299-Luc tumors growing in the lung of mice. **Panel II**, immunohistochemical image of the corresponding antibody isotype control.

To examine the therapeutic potential of iNOP-7-PLK1 siRNA *in vivo*, we developed H1299 NSCLC cells to stably express high levels of firefly luciferase (H1299-Luc) to allow for the measurement of tumor growth in an orthotopic NSCLC mouse model (Figure 5A, panel I). Importantly, lung tumors in this model grow in a microenvironment which closely mimics the human setting. Seven days post-tumor cell inoculation, tumors could be detected in the lungs of mice as evidenced by a strong bioluminescent signal (Figure 5A, panel II). The bioluminescent signal increased over time, and by day 20 diffuse lung tumor growth was observed. To assess whether iNOP-7 could deliver clinically-relevant amounts of siRNA to growing lung tumors, mice (15 days post-tumor cell inoculation) were administered systemically with iNOP-7 complexed to siRNA which was labeled with a near-infrared dye (AlexaFluor 647). Mice injected with PBS or fluorescent siRNA alone served as controls. Four hours post-injection, lungs (including lung tumor), liver and spleen were harvested, and *ex-vivo* fluorescent intensity measured. As expected, no fluorescence was detected in mice injected with fluorescent siRNA alone, indicating that the siRNA was eliminated from the body (Figure 5B). In contrast, mice injected with iNOP-7-siRNA complexes showed strong fluorescence in the lung and the surrounding tumor tissue as well as the liver and spleen (Figure 5B). Micro-CT imaging confirmed the presence of tumors growing within the lung of mice treated with iNOP-7-fluorescent siRNA (Figure 5C, panel I). Moreover, confocal microscopy confirmed the presence of fluorescent siRNA within tumor, liver and spleen tissue following delivery with iNOP-7 (Figure 5C, panels II-IV). Importantly, we also showed that human lung tumors growing in the mouse lung expressed our target protein PLK1 (Figure 5D). Finally, to confirm that iNOP-7 was non-toxic, we treated mice with iNOP-7 (8:1 w/w) or PBS (control). Twenty-four hours post-injection, lung, liver and spleen were harvested and gross histology assessed by hematoxylin and eosin staining. No obvious change in histology in the lung, liver or spleen was observed in mice treated with iNOP-7 when compared to controls (Supplementary Figure 5). Collectively, these results show for the first time that iNOP-7 can deliver siRNA to growing lung tumors in a cellular microenvironment which recapitulates many of the features observed in the human clinical setting.

### iNOP-7-PLK1 siRNA reduces NSCLC growth *in vivo*

**Figure 6:**
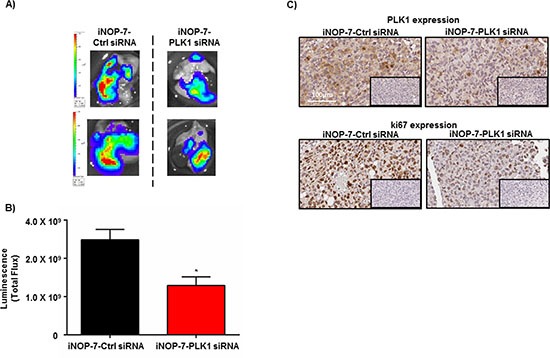
Effect of iNOP-7-PLK1 siRNA on NSCLC growth *in vivo* **(A)** *Ex-vivo* images of whole lungs from 2 individual mice collected after treatment with iNOP-7-control (Ctrl) siRNA or iNOP-7-PLK1 siRNA. **(B)** A Graph demonstrating a 50% decrease in total whole lung bioluminescence [measured as total flux (photons/sec)] from each individual mouse treated with iNOP-7-PLK1 siRNA (X2 week / total of 2 weeks) vs. mice treated with iNOP-7-control (Ctrl) siRNA, n = 3-4 per treatment group; bars, mean ± SE. *p < 0.01. **(C)** Immunohistochemical images showing a decrease in PLK1 and ki67 expression in sections of tumors collected from mice treated with iNOP-7-PLK1 siRNA vs. mice treated with iNOP-7-control (Ctrl) siRNA. Inserts represent the corresponding negative control.

Given that the orthotopically implanted human NSCLC cells grow rapidly into tumors in the mouse lung and express high levels of PLK1, we next assessed the therapeutic potential of iNOP-7-PLK1 siRNA *in vivo*. Mice were orthotopically implanted with H1299-Luc cells into the lung, and 7 days post-tumor cell inoculation and prior to treatment, luminescence was measured in all mice which confirmed the presence of lung tumors of similar size (Supplementary Figure 6A). Mice were randomized into treatment groups (n=3-4 mice per group) and administered with low amounts of iNOP-7-PLK1 siRNA (1.25 mg/kg) or iNOP-7-control (Ctrl) siRNA (1.25 mg/kg) twice weekly for 2 weeks. Body weight was monitored throughout the treatment period, and no decrease was observed in mice treated with iNOP-7-siRNA (Supplementary Figure 6B). At day 20, whole lungs were harvested and *ex-vivo* luminescence measured. Mice treated with iNOP-7-PLK1 siRNA had a 50% (*p* < 0.01) decrease in luminescence in whole lungs when compared to controls (Figure 6A and 6B). Immunohistochemical staining for PLK1 expression in tumor tissue indicated there was a decrease in PLK1 levels in areas of lung tumors collected from mice treated with iNOP-7-PLK1 siRNA when compared to controls (Figure 6C). To confirm the decrease in tumor growth, tumor tissue sections were stained with the proliferative marker ki67. Notably, there was a decrease in ki67 expression in areas of lung tumors from the mice treated with iNOP-7-PLK1 siRNA (Figure 6C), thus providing evidence that systemic administration of iNOP-7-PLK1 siRNA reduced NSCLC cell proliferation *in vivo*. The ability of iNOP-7-PLK1 siRNA to reduce NSCLC tumor growth *in vivo* was further illustrated in a mouse model of experimental metastases, where H1299-Luc NSCLC cells were injected systemically (tail vein) into mice leading to the colonization and growth of NSCLC cells in the lungs and other sites of the body (i.e. lymph nodes). Prior to treatment (day 21 post-injection) with iNOP-7-PLK1 siRNA, all mice with similar whole body tumor luminescence (Supplementary Figure 7A) were randomized into treatment groups (n = 5-6 mice / group), and administered systemically with iNOP-7-PLK1 siRNA (1.25 mg/kg) or iNOP-7-control (Ctrl) siRNA (1.25 mg/kg) (days 23, 26, 29, 32 and 34). Whole body luminescence was measured once during the treatment period (day 28) and at post-treatment (day 35). Notably, mice treated with iNOP-7 PLK1 siRNA had a decrease in whole body luminescence during the treatment period (day 28; 54% decrease in luminescence) and at the end of the treatment (day 35; 62% decrease in luminescence) when compared to controls (Supplementary Figure 7B). Collectively, these findings show that iNOP-7-PLK1 siRNA is an effective therapeutic tool to reduce NSCLC tumor growth *in vivo*.

## DISCUSSION

PLK1 is recognized as a drug target for cancer therapeutics, and intense research has been devoted to the development of chemical molecules which inhibit the function of PLK1. However, off-target side effects as a result of inhibiting the function of other closely related protein kinases or poor tumor bioavailability limit their clinical potential. Here, we report for the first time the ability of iNOP-7 nanoparticles to self-assemble and deliver siRNA with high efficiency to NSCLC cells. When complexed to PLK1 siRNA, iNOP-7 nanoparticles potently silenced PLK1 expression in NSCLC cells, markedly reduced cell proliferation and induced apoptosis. Finally, iNOP-7-PLK1 siRNA injected systemically at clinically-relevant doses (1.25mg/kg) reduced NSCLC tumor growth in an orthotopic lung cancer mouse model.

PLK1, like other polo-like kinase (PLK) protein family members, has been reported to play diverse but critical roles in regulating the cell cycle and/or cellular response to DNA damage [5]. PLK1 has been the most extensively studied PLK protein and an increasing number of clinical studies describe high levels of PLK1 in tumor cells, and a correlation to aggressive tumor growth and poor survival [24–28]. For instance, a recent study reported that human NSCLC tumors collected after surgical resection express high levels of PLK1 compared to the surrounding normal tissue, and high PLK1 expression in these tumors was associated with advanced stage disease and lymph node metastases [6]. We demonstrated that multiple NSCLC cell lines express high levels of PLK1 when compared to normal human non-tumorigenic lung fibroblasts. Moreover, our data is consistent with previous reports showing that inhibition of PLK1 using either chemical inhibitors or RNAi induces mitotic arrest in tumor cells which leads to a reduction in cell proliferation and increased cell death [6, 10, 12, 27, 29]. In accordance with other studies, we also showed that potent inhibition of PLK1 using siRNA did not induce cell death in normal non-tumorigenic cells. This was despite both NSCLC cells and lung fibroblasts having not too dissimilar doubling time *in vitro*. Interestingly, studies have reported that cells with KRAS and/ or p53 mutations are hypersensitive to PLK1 inhibition when compared to their wild-type counterparts [29, 30]. Notably, our NSCLC cell lines had mutations in either KRAS or p53, both of which are common in NSCLC. Therefore, it is possible that NSCLC cells with mutations in KRAS or p53 may be highly susceptible to therapies which specifically inhibit PLK1 expression or activity.

RNAi based therapeutics is emerging as an exciting novel approach to treat human disease. However, a major challenge to the field has been the development of highly effective delivery vehicles for RNAi molecules to enter target cells. In this study, we show for the first time that iNOP-7 can complex and deliver siRNA with high efficacy to NSCLC cells. In support of our previous reports [19, 20, 22] we showed that iNOP-7 was non-toxic *in vitro* and *in vivo*. Notably, iNOP-7 was able to rapidly and efficiently complex with siRNA targeting PLK1 and deliver it to NSCLC cells. This resulted in the potent knockdown of PLK1 expression, a marked reduction in cell growth and a potent increase in apoptosis. Based on the strong *in vitro* data which supported the use of iNOP-7-PLK1 siRNA to reduce NSCLC cell growth, we next investigated the potential of iNOP-7-PLK1 siRNA to reduce lung tumor growth in a clinically-relevant orthotopic NSCLC mouse model. In this model human NSCLC cells when implanted into the lung, form initially as a solitary tumor, and over time disseminate into both lungs and the surrounding lymph nodes. Notably, this pattern of tumor growth and development closely mimics the human scenario [31]. Furthermore, it has been reported that orthotopic lung tumors respond to chemotherapeutic drugs in a similar manner to what is observed in the clinical setting [31]. Importantly, we showed that systemic administration of iNOP-7-siRNA was able to deliver siRNA to the lungs and surrounding tumors. Moreover, injection of clinically acceptable amounts of iNOP-7 PLK1 siRNA decreased lung tumor bioluminescence by 50% (indication of reduced tumor burden) when compared to mice treated with iNOP-7 control siRNA. In support of this finding we also found a decrease in the expression of the proliferation marker ki67 in lung tumor tissue. These data are in accordance with our *in vitro* studies which demonstrated the strong anti-proliferative effect in NSCLC cells upon silencing PLK1 expression using iNOP-7-siRNA. It should be noted, that iNOP-7 also delivered high amounts of siRNA to the liver of mice with orthotopic lung tumors. This is not unexpected given our previous studies showing biodistribution of iNOP-7 to the liver [19, 21, 22]. This is likely attributable to the fact that iNOP-7 was not modified to specifically target tumor cells. Therefore, it is likely that upon systemic administration a percentage of iNOP-7-siRNA was able to enter lung tumors due to passive targeting via the enhanced permeability and retention effect. Support for this is provided by immunohistochemical staining of our orthotopic NSCLC tumors, which identified CD31-positive blood vessels within the tumor (results not shown). Collectively, to the best of our knowledge this is the first study to demonstrate the systemic delivery of low amounts of siRNA to mice with orthotopic NSCLC lung tumors using modified-dendrimer nanoparticles.

iNOP-7 nanoparticles possess unique physicochemical properties which give them several advantages when compared to other dendrimer and polymer-based nanoparticle systems. Namely, iNOP-7 is composed from a naturally occurring amino acid, lysine. This feature provides iNOP-7 with a highly favourable toxicity profile when compared to other nanoparticles with a similar shape and/or size such as poly(amidoamine) PAMAM dendrimers, which despite their high transfection efficiency suffer from toxic and immuostimulatory side-effects [32]. In addition, the surface of iNOP-7 has been modified via the chemical conjugation of seven oleic acid lipid chains to increase its hydrophobicity and ability to interact with cell membranes, thus increasing its cell uptake. This translates to reduced amounts of iNOP-7 being needed to deliver siRNA with high efficiency into cells. Moreover, the large surface volume of iNOP-7 allows for highly efficient and rapid interactions with different types of therapeutic drug molecules including, siRNA, DNA or conventional chemotherapeutics. Finally, the surface of iNOP-7 can be easily functionalized to contain a number of different moieties to improve drug release or target specific cell types. Recently, we developed a next-generation iNOP nanoparticle which contains a reducible (disulfide bond) spacer on its surface to improve the release kinetics of siRNA once in contact with a cellular microenvironment [33]. Future studies designed to modify the surface of iNOP-7 to contain tumor cell-targeting moieties in an effort to improve the amount of siRNA specifically delivered to lung tumor cells are being undertaken in our laboratory.

In conclusion, this study has provided the first evidence that iNOP-7 nanoparticles deliver siRNA to tumor cells both *in vitro* and *in vivo*. Furthermore, iNOP-7 when complexed to PLK1 siRNA potently silences PLK1 expression in NSCLC cells which reduces their growth in a clinically-relevant orthotopic NSCLC mouse model. These findings highlight the potential of iNOP-7-PLK1 siRNA as a novel therapeutic strategy for the treatment of NSCLC as well as other cancers which aberrantly express PLK1.

## MATERIALS AND METHODS

### Cell culture

Human H460, Calu-6, H1299, H441 and H1975 NSCLC cell lines, and MRC-5 human embryonic normal lung fibroblasts were obtained from American Tissue Culture Collection (Manassas, VA, USA). Cells were maintained in Roswell Park Memorial Institute (RPMI) medium (H460, H441, H1975) or Dulbecco's Modified Eagle's Medium (DMEM) medium (Calu-6, H1299) or MEM (MRC-5) containing 10% fetal calf serum (FCS) and 2 nmol/L L-glutamine (MRC-5 cells were additionally supplemented with 1 mM sodium pyruvate, 0.1 mM non-essential amino acids and 1.5g/L sodium bicarbonate) at 37°C in a humidified atmosphere with 5% CO_2_. Cells were routinely screened and found to be free of mycoplasma.

### Real-time quantitative PCR (qPCR)

The expression of PLK1 mRNA in NSCLC and MRC-5 cells was examined using qPCR. Total RNA was isolated as previously described [34–36]. qPCR was performed using the Applied Biosystems high capacity cDNA reverse transcription and SYBR green kits as described [34–36]. PLK1 mRNA expression was measured using the Hs_PLK1_1_SG Quantitect primer assay (Qiagen, Melbourne, Vic, Australia). All data were normalized to the housekeeping gene *β_2_*-microglobulin** using the β_2_-Microglobulin QuantiTect Primer Assay, (Qiagen, Melbourne, Vic, Australia).

### Western blotting

Western blot analyses on whole cell lysates were performed using the following antibodies as described previously [34–36]: PLK1 (208G4) rabbit monoclonal antibody (Cell Signaling Technology, USA), cleaved PARP (Asp 214) (D64E10) rabbit monoclonal antibody (Cell Signaling Technology, USA), cleaved caspase 3 (Asp 175) polyclonal antibody (Cell Signaling Technology, USA) and Glyceraldehyde-3-phosphate dehydrogenase (GAPDH; clone 6C5; Abcam).

### siRNA transfection

NSCLC or MRC-5 cells were transfected with siRNA (1-100 nM) complexed to lipofectamine 2000 (L2K) (Invitrogen, Waltham, MA, USA) or to iNOP-7 [1:1–8:1 (w/w) with siRNA] as described [19, 34]. Two individual PLK1 siRNA sequences targeted against different regions of the gene (designated PLK1 siRNA-1 and PLK1 siRNA-2), were used. PLK1 siRNA-1 [On-Target Plus (J-003290-09), GE healthcare]: target sequence: GCACAUACCGCCUGAGUCU; PLK1 siRNA-2 (On-Target plus (J-003290-10), GE healthcare], target sequence: CCACCAAGGUUUUCGAUUG. Non-silencing siRNA (GE healthcare) and AlexaFluor-488 and AlexaFluor-647 siRNA (Qiagen, Melbourne, Vic, Australia) were used as controls.

### Cell proliferation assay

Following siRNA (1-100 nM) transfection (72h), cell metabolic activity was measured using resazurin solution (Alamar Blue) (597 mmol resazurin, 67 mmol methylene blue, 1 mmol potassium hexacyanoferrate (III), 1 mmol potassium hexacyanoferrate (II) trihydrate in PBS) and fluorescence measured using a Victor2 plate reader (Perkin Elmer, USA) as described [35]. Cells transfected with non-silencing siRNA served as controls.

### Apoptosis assays

NSCLC or MRC-5 cells were transfected with PLK1 siRNA (1-100 nM) or control (non-functional) siRNA. 24-72h post-transfection apoptosis was determined by Annexin V-FITC staining using flow cytometry and by measurement of caspase 3/7 activity using the Caspase-Glo 3/7 assay as previously described [36–38].

### Cell cycle analysis

NSCLC cells were transfected with PLK1 or control siRNA (5-100 nM), and 48h post-transfection, cells were harvested (floating and adherent), washed with PBS and fixed with 80% ethanol for at least 24h at 4°C. The fixed cells were then stained with a solution containing 50 μg/ml propidium iodide, and 2 μg/ml DNase-free RNase for 30 min at 37°C in the dark. DNA content was measured by a FACSCalibur flow cytometer (BD Biosciences).

### siRNA binding and gel retardation assay

To determine the amount of iNOP-7 needed to fully self-assemble with siRNA, increasing amounts of iNOP-7 (w/w ratio with siRNA) were complexed to a fixed amount of siRNA (400 ng) in OptiMEM I reduced serum culture medium for 20 min at room temperature. The complexes were then electrophoresed and analysed as described [34].

### Toxicity analysis

To assess the toxicity of iNOP-7, H460 and Calu-6 NSCLC cells as well as normal human lung fibroblasts (MRC-5) were plated into 6 well tissue culture plates 16h before transfection. The following day, cells were treated with iNOP-7 alone or complexed to non-silencing (control) siRNA [8:1(w/w)]. 24h post-treatment cell viability was measured using a colorimetric cell viability assay (Dojindo Cell Counting Kit) as described [34].

### siRNA cell uptake *in vitro*

To measure cell uptake of siRNA complexed to iNOP-7, H460 and Calu-6 NSCLC cells were plated into 6-well culture plates (6 × 10^4^) and the following day, transfected with iNOP-7 [8:1 (w/w)]-siRNA labelled with AlexaFluor-488 (green) at 37°C. Twenty four hours post-transfection the cells were washed 3 times in warm phosphate buffered saline (PBS) and harvested. The fluorescent intensity within the cells was measured using the FACSCalibur flow cytometer (BD Biosciences). Cells treated with AlexaFluor-488-labeled siRNA alone or complexed to lipofectamine 2000 served as controls. To observe the intracellular pattern of siRNA complexed to iNOP-7, cells were plated (3 × 10^3^) into 4-well Nunc Lab-Tek™ tissue culture chamber slides. The following day cells were transfected with iNOP-7 complexed to AlexaFluor-488-labeled siRNA. Cells transfected with fluorescent siRNA alone, or siRNA complexed to lipofectamine 2000 served as controls. 24h post-transfection cells were fixed using fresh 4% paraformaldehyde and stained with the Image-iT LIVE Plasma membrane and nuclear staining kit (Life Technologies, Vic, Australia). The slides were mounted with VECTASHIELD® anti-fade mounting media and siRNA uptake was visualized using an Olympus FluoView™ FV1000 confocal microscope. To examine the release of siRNA from lysosomes/endosomes within the cell cytoplasm, H460 NSCLC cells were transfected with iNOP-7 complexed to AlexaFluor-488-labeled siRNA. One, four and eighteen hours post-transfection cells were stained with 1μM Lysotracker® (Life Technologies, Australia), fixed and imaged on a confocal microscope as described above.

### Generation of stably expressing luciferase NSCLC cells

H1299 NSCLC cells stably expressing firefly luciferase (designated H1299-Luc) were generated via retroviral delivery of the SFG-_NES_ (SFG-Ntp backbone with nuclear export signal) TGL triple-modality reporter construct, harboring the herpes virus 1 thymidine kinase gene, Aequorea victoria green fluorescent protein (GFP) gene and the *Photinus pyralis* (firefly) luciferase gene (construct was a kind gift from Dr Vladimir Ponomarev, Memorial Sloan Kettering Cancer Centre, USA) as previously described [39]. A high GFP expressing cell population was then selected by fluorescence-activated cell sorting. High and stable expression of luciferase was confirmed using the IVIS Imaging system as described [35].

### Orthotopic NSCLC mouse model

H1299-Luc NSCLC cells (5 × 10^5^) were injected orthotopically into the left lung of 8-week old female SCID-Beige mice as described with minor modifications [40]. All animal experiments were approved by the Animal Ethics Committee, UNSW Australia (ACEC no. 13/130B). Briefly, mice were anaesthetized using isoflurane. The chest was swabbed with 70% alcohol and a small skin incision to the left chest wall (approximately 5 mm in length) was made approximately 5 mm tail side from the scapula. Skin fat and muscles were separated and on observing the left lung motion through the pleura, a 29 gauge needle attached to a 0.5 ml insulin syringe was directly injected through the intercostal space into the lung to a depth of approximately 3 mm. Tumor cells (5 × 10^5^) were suspended in 30 μl of growth factor-reduced Matrigel (BD Biosciences, NSW, Australia) to minimize leakage from the lung and injected directly into the lung parenchyma. The skin incision was closed with a surgical skin clip. After confirming that the animals had recovered and possessed stable spontaneous respiration, they were returned to their cages. Seven-to-ten days post-tumor cell injection, growing lung tumors were confirmed using micro-CT analysis or bioluminescent imaging.

### Micro-CT analysis

Micro-computed tomography (CT) scanning was used to confirm the presence of growing tumors within the lungs of mice. The micro-CT data were acquired using an Inveon system (Siemens) at 71.88 pixel size, 220 projections, 250 ms integration time, 50 keV photon energy and and 450 μA current. Image data were evaluated using the Inveon software package (Siemens). Tumor margins were identified by contrast thresholding which allowed the tumor margins (soft tissue density) to be defined against the surrounding lung (air density).

### Luciferase detection

Luciferase-expressing NSCLC cells were visualized *in vivo* by intraperitoneal injection of 150 mg kg^−1^ D-luciferin (Gold Biotechnology, St Louis, MO, USA). Animals were anaesthetized, placed inside the IVIS Imaging system specimen chamber and imaged approximately 10 min post-injection. For *ex vivo* imaging, 150 mg kg^−1^ D-luciferin was injected intraperitoneal before necropsy. Animals were killed and their lungs and tumor harvested and covered with D-luciferin as described [35]. Bioluminescent images were digitized and electronically displayed as a pseudocolor overlay onto a gray scale image on the LIVINGIMAGE software. Colored scale bars on figures indicate the amount of bioluminescence as measured in photons/second (p/sec).

### siRNA biodistribution *in vivo*

To assess the biodistribution of siRNA complexed to iNOP-7 *in vivo*, mice were orthotopically injected with H1299-Luc NSCLC cells. Fifthteen-days post-injection, mice were administered with iNOP-7 complexed to AlexaFluor-647 (Red) siRNA (30 μg) via the tail vein. Mice injected with PBS or fluorescent siRNA alone served as controls. Four and 24h post-administration mice were killed and lungs, tumor, liver and spleen harvested and near-infrared fluorescence measured using the IVIS Imaging system. After *ex-vivo* images were captured, organs were snap frozen in OCT embedding media. Frozen sections (10 μm) of the tumor, liver and spleen tissue were then placed onto saline coated histological slides and mounted with VECTASHIELD® anti-fade mounting media with DAPI. siRNA uptake was visualized using an Olympus FluoView™ FV1000 confocal microscope.

### iNOP-7-PLK1 siRNA administration *in vivo*

To examine the therapeutic potential of iNOP-7-PLK1 siRNA *in vivo*. H1299-Luc cells were injected into the lung of mice. Seven-days post-tumor cell inoculation, the presence of growing lung tumors was confirmed by bioluminescent imaging. Mice were then randomized into treatment groups and administered with iNOP-7 complexed to PLK1 siRNA (1.25 mg/kg) via the tail vein, twice weekly for 2 weeks. Mice injected with non-functional siRNA served as controls. At the end of the experiment (Day 20) mice were killed and total lungs harvested and *ex-vivo* bioluminescence measured using the IVIS bioimaging system. Whole lungs were then fixed in 4% paraformaldehyde for histological analysis.

### Immunohistochemistry

Immunohistochemistry was performed on paraffin-embedded mouse lung tumor tissue sections to measure the expression of the proliferation marker ki67 as well as PLK1. Briefly, tissue sections were incubated with either a mouse monoclonal rabbit ki67 antibody (clone MIB-1, Dako) or a monoclonal rabbit PLK1 antibody (Cell Signaling Technology). 3,3′;-diaminobenzidine (DAB) was used as a substrate for the peroxidase reaction and hematoxylin as the counterstain. Omission of the primary antibody, or incubating tissue sections with immunoglobulin G antibodies at a concentration equivalent to the primary antibody served as controls. Slides were scanned using an Aperio ScanScope XT Slide Scanner (Aperio, Vista, CA, USA). Images were analyzed using the ImageScope software (Aperio).

### Statistical analysis

Statistical analyses were performed using the GraphPad 6 Prism program. Results are expressed as means of at least 3 independent experiments ± standard error of the mean (SEM) (error bars). ANOVA or Unpaired two-tailed Student's *t* tests followed by Tukey's or Dunnet post tests were used to determine the statistical differences between various experimental and control groups, with *p* < 0.05 considered statistically significant.

## SUPPLEMENTARY FIGURES



## Abbreviations

NSCLC: Non-small cell lung cancer
iNOP-7: Interfering nanoparticle-7
RNAi: RNA interference
PLK1: Polo-like kinase 1
siRNA: Small-interfering RNA
