# Supplementary material for: Therapeutic targeting of polo-like kinase 1 using RNA-interfering nanoparticles (iNOPs) for the treatment of non-small cell lung cancer
Source: Oncotarget. 2015 Jan 6;6(14):12020–34. doi: 10.18632/oncotarget.2664 (PMC4494920; doi:10.18632/oncotarget.2664)
Supplement: Supplementary file 1 [file oncotarget-06-12020-s001.pdf]

SUPPLEMENTARY FIGURES

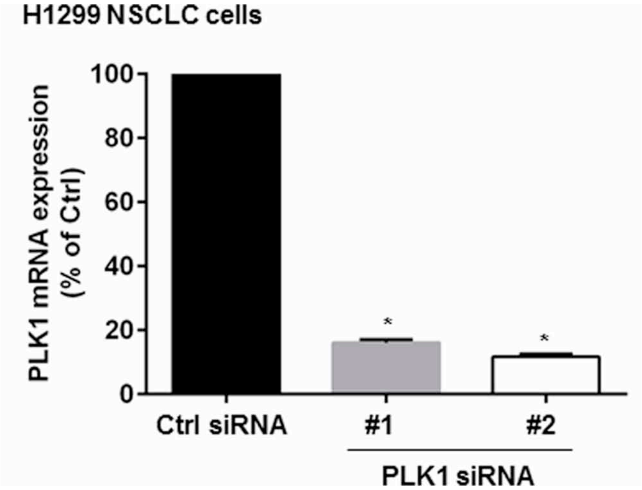

Supplementary Figure S1: Gene silencing activity of PLK1 siRNAs in NSCLC cells. A graph showing 2 individual siRNAs targeted against the PLK1 gene are able to potently silence PLK1 mRNA levels > 80% in H1299 cells 48h post-transfection using lipofectamine 2000 as the delivery vehicle, n = 3; bars, mean ± SE. *$p < 0.001$.

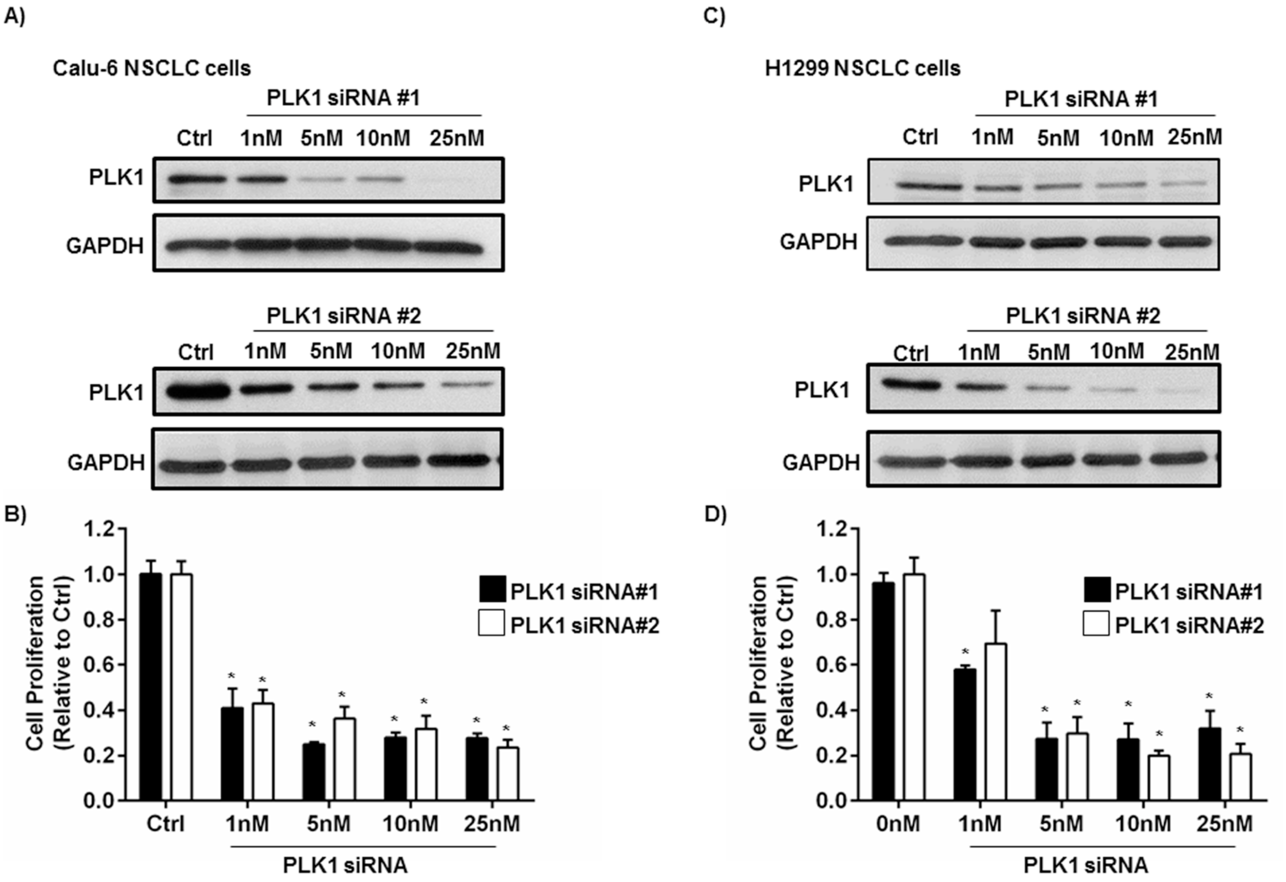

Supplementary Figure S2: Effect of silencing PLK1 expression using 2 different PLK1 siRNAs on H1299 and Calu-6 NSCLC cell proliferation. (A and C) Representative western blots demonstrating a dose-dependent decrease in PLK1 protein expression in Calu-6 and H1299 NSCLC cells 72h post-treatment with increasing concentrations of PLK1 siRNA #1 or PLK1 siRNA #2 (1-25 nM). GAPDH was used as a protein loading control. **(B and D)** Graphs showing a significant decrease in cell proliferation in Calu-6 and H1299 cells, 72h post-treatment with increasing concentrations of PLK1 siRNA #1 or PLK1 siRNA #2 (1-25 nM), n = 3; bars, mean ± SE. *$p < 0.01$.

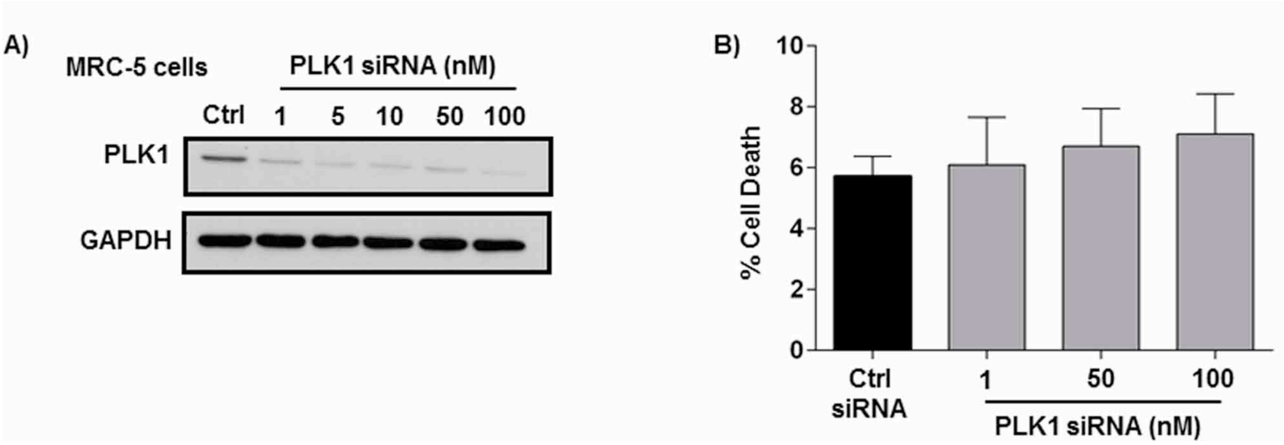

Supplementary Figure S3: Effect of PLK1 siRNA on normal human lung fibroblast cell viability. **(A)** Representative western blot showing potent knockdown of PLK1 protein expression in normal human lung fibroblasts (MRC-5) 72h post-treatment with increasing concentrations of PLK1 siRNA (1-100 nM) complexed to lipofectamine 2000. Cells treated with non-functional (Ctrl) siRNA served as controls. GAPDH was used as a protein loading control, n = 3. **(B)** A graph showing no significant effect on cell death as measured by annexin V staining and flow cytometry in normal human lung fibroblasts (MRC-5) 48h post-treatment with increasing concentrations of PLK1 siRNA (1-100 nM). Cells treated with non-functional (Ctrl) siRNA served as controls, n = 3; bars, mean ± SE.

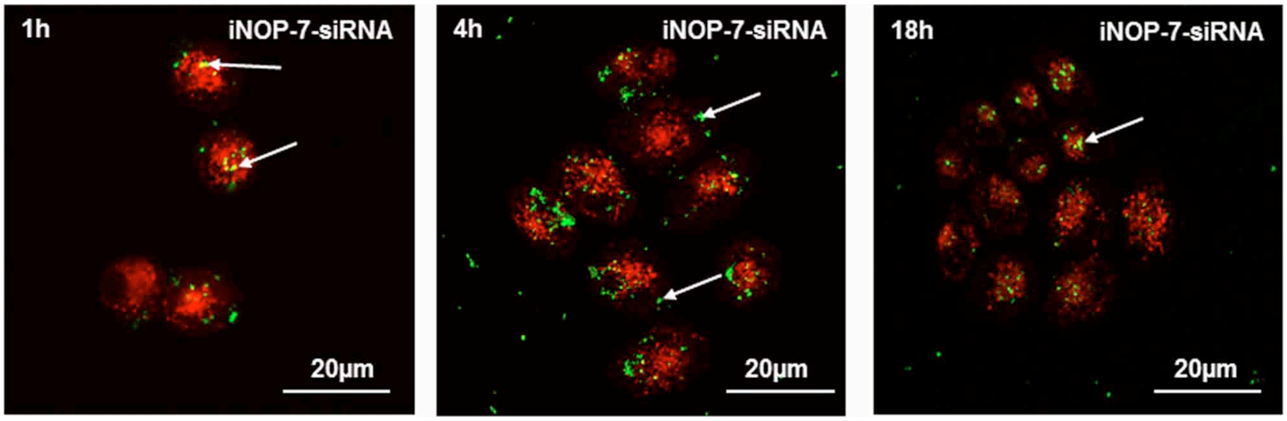

Supplementary Figure S4: Localization of siRNA within NSCLC cells *in vitro*. Representative confocal images showing the localization of fluorescent siRNA (green) in NSCLC cells when complexed to iNOP-7 (8:1 w/w). Cells were fixed and stained with lysotracker Red (red) to stain for endosomes and lysosomes at 1h, 4h, and 18h post-treatment with iNOP-7-fluorescent siRNA. Co-localization with endosomes / lysosomes can be observed 1h post-treatment (white arrow marks co-localization, yellow dots). At 4h and 18h there is no co-localization with endosomes / lysosomes indicating effective release of siRNA into the cytosol (white arrows mark siRNA).

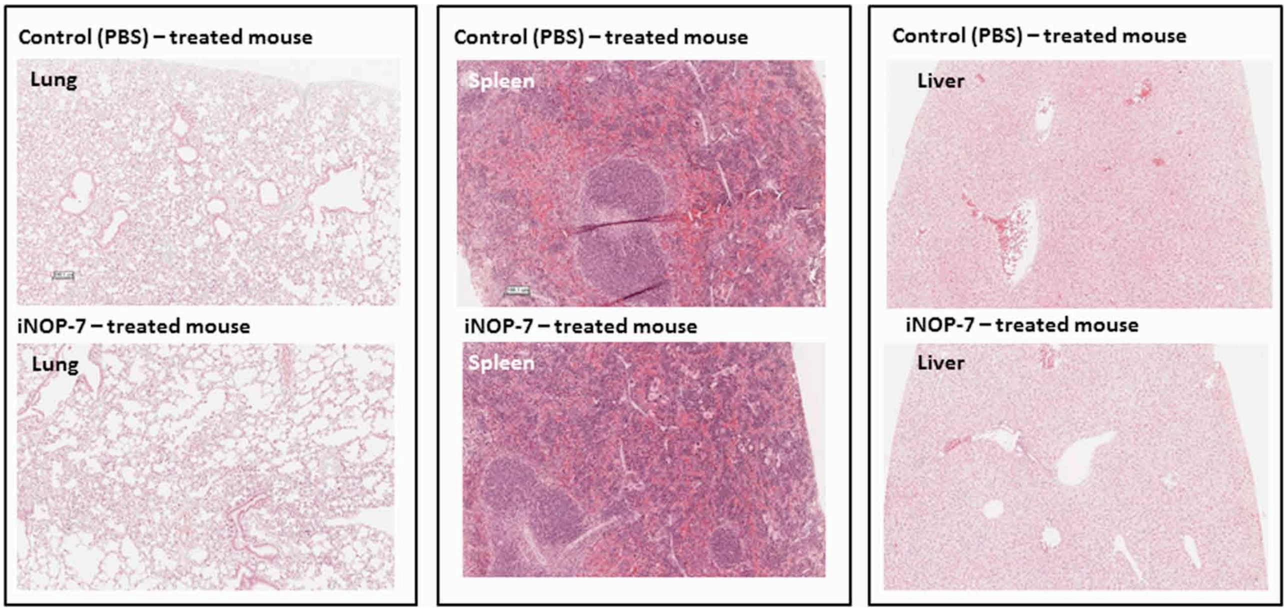

Supplementary Figure S5: Effect of iNOP-7 on the morphology of lung, liver and spleen of immunocompetent mice. Representative images of hematoxylin and eosin (H&E) staining of lung, liver and spleen from mice treated systemically with iNOP-7 alone (8:1 w/w). 24h post-treatment lung, liver and spleens were harvested and gross morphology assessed by H&E staining and examination under a light microscope. Mice treated with PBS served as controls.

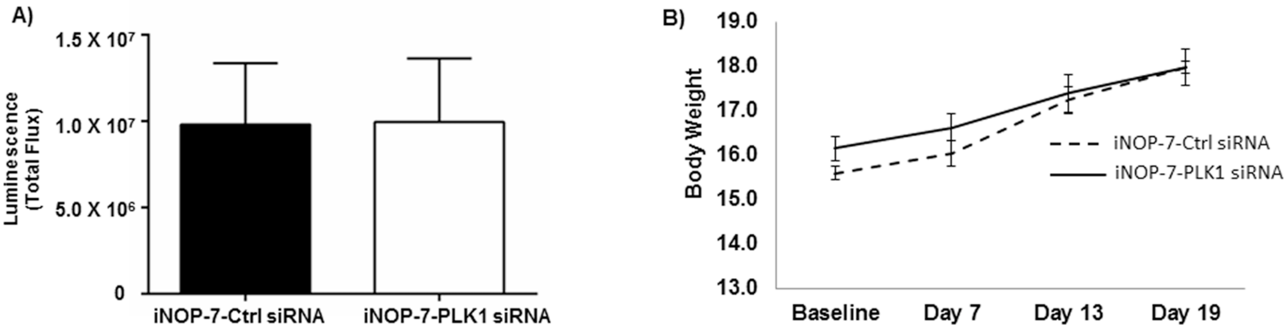

Supplementary Figure S6: Lung bioluminescence in mice with orthotopic tumors prior to treatment with iNOP-7-PLK1 siRNA and total mouse body weight during iNOP-7-siRNA treatment. (A) Similar bioluminescent activity is observed in the lungs of mice with orthotopic NSCLC tumors, just prior to treatment with either iNOP-7-PLK1 siRNA or iNOP-7 non-functional (Ctrl) siRNA, n = 3-4 mice / group; bars, mean ± SE. **(B)** The total body weight of mice with orthotopic NSCLC tumors was measured during the 2 week treatment period with iNOP-7-PLK1 siRNA or iNOP-7-non-functional (Ctrl) siRNA, n = 3-4 mice / group.

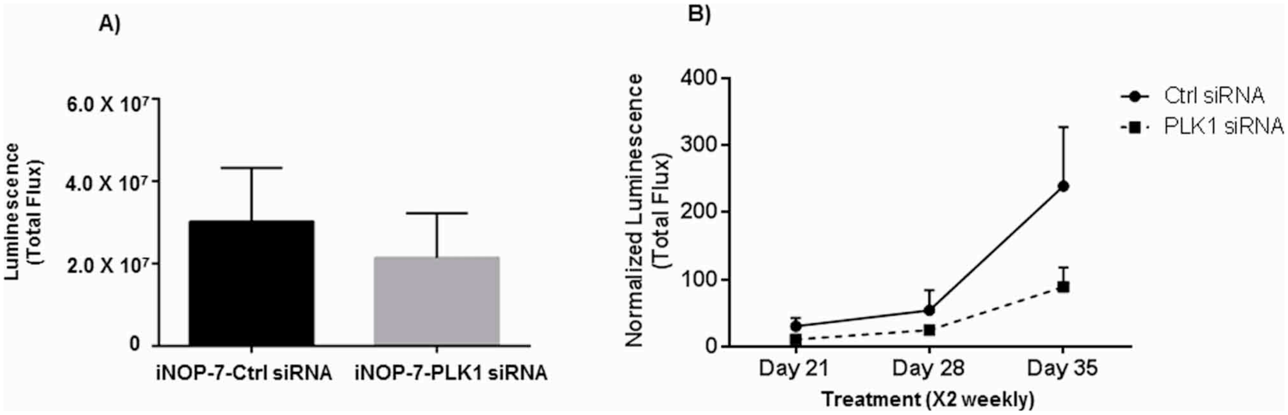

Supplementary Figure S7: Effect of iNOP-7-PLK1 siRNA on experimental metastatic tumor growth in mice. **(A)** Similar levels of total luminescence was observed in mice 21 days post-injection (via the tail vein) with $2X10^6$ H1299-Luc cells prior to treatment with iNOP-7-PLK1 siRNA or iNOP-7-non-functional (Ctrl) siRNA, n = 6 mice/group; bars, mean ± SE. **(B)** A graph showing a decrease in whole body total luminescence in mice treated with iNOP-7-PLK1 siRNA vs. mice treated with iNOP-7-control (Ctrl) siRNA during the treatment period [day 23-35] (X2 weekly, total of 5 treatments, n = 6 mice / group)].
